# Determining Effects of Winter Weather Conditions on Adult *Amblyomma americanum* (Acari: Ixodidae) Survival in Connecticut and Maine, USA

**DOI:** 10.3390/insects11010013

**Published:** 2019-12-21

**Authors:** Megan A. Linske, Scott C. Williams, Kirby C. Stafford, Charles B. Lubelczyk, Elizabeth F. Henderson, Margret Welch, Peter D. Teel

**Affiliations:** 1 Department of Entomology, Center for Vector Biology and Zoonotic Diseases, The Connecticut Agricultural Experiment Station, P.O. Box 1106, New Haven, CT 06504, USA; megan.linske@ct.gov (M.A.L.);; 2 Department of Forestry and Horticulture, Center for Vector Biology and Zoonotic Diseases, The Connecticut Agricultural Experiment Station, P.O. Box 1106, New Haven, CT 06504, USA; 3 Vector-borne Disease Laboratory, Maine Medical Center Research Institute, 81 Research Drive, Scarborough, ME 04074, USA; lubelc@mmc.org (C.B.L.); ehenderson@mmc.org (E.F.H.); margret.welch@maine.edu (M.W.); 4 Department of Entomology, Texas A&M University, TAMU 2475, College Station, TX 77843, USA; pteel@tamu.edu

**Keywords:** *Amblyomma americanum*, climate change, overwintering survival

## Abstract

The lone star tick (*Amblyomma americanum* L.) is native to the United States, with its primary range encompassing the Southeast and portions of the Midwest. It is an aggressive ectoparasite that actively seeks out hosts through detection of carbon dioxide and vibrations and can transfer ehrlichiosis-causing bacteria as well as a carbohydrate that causes alpha-gal syndrome (red meat allergy) in humans. It has become of increasing concern as its range has recently expanded into coastal regions of the Northeast. Historically, harsh northeastern winter weather conditions made these areas inhospitable for *A. americanum* survival, but a warming climate coupled with increased host availability seem to have facilitated their range expansion. We developed a study to observe the effects of weather conditions on adult *A. americanum* overwintering survival. The study was conducted over three years in Connecticut and Maine. Ground-level conditions were manipulated to determine the effects of differing combinations of natural insulative barriers (leaf litter and snow accumulation) on adult *A. americanum* survival. We determined that there was a significant difference in survival between the two states, between years in Maine, and between sexes within Connecticut. However, presence or absence of snow and/or leaf litter had no impact on survival. Overall, we found a positive correlation between mean hourly temperature and adult survival in Maine, where temperatures were consistently below freezing. The results of this study can be included in an adaptive, predictive analytic model to accommodate the expected fluctuations and range expansion of *A. americanum* that will most likely accompany an increase in temperatures throughout the Northeast.

## 1. Introduction

Due to factors such as habitat modifications, altered host dynamics, and climate change, existing and emerging ticks and tick-borne diseases are increasingly more prevalent [1,2]. *Ixodes* ticks, such as blacklegged ticks (*Ixodes scapularis* Say), have not only expanded their range, but also significantly increased in density. Their expansion is due, in part, to chronically overabundant host species such as white-tailed deer (*Odocoileus virginianus* Zimmermann) [3,4] as well as a longer questing season and reduced mortality due to milder weather [5,6,7]. In addition to native tick species range expansion, there are also introductions of exotic species to contend with. The Asian longhorned tick (*Haemaphysalis longicornis* Neumann) originated from eastern Asia, but populations are now established in Australia, New Zealand, the Pacific Islands, and most recently the United States [8]. While their susceptibility to dry conditions might limit their range [9], their success in warmer climates [10,11], ability to survive cold winters [12], and the fact that they are parthenogenetic [8,13,14] makes them a significant concern for human, wildlife, and livestock health. But of equal concern are native ticks that are now expanding their range and establishing populations in areas where they did not exist previously, just as the lone star tick (*Amblyomma americanum* L.) is doing in coastal areas of the Northeast.

*Amblyomma americanum* has historically inhabited the Southeast and parts of the Midwest [15]. There had been limited northward migration presumably due to harsher, colder winters, but this climatic barrier appears to have been breached along coastal New York and New England in recent years. Breeding populations of *A. americanum* are currently established throughout Long Island, New York [16,17] as well as Prudence Island and smaller islands in Narragansett Bay, Rhode Island [17,18], and have been reported on Cape Cod and Martha’s Vineyard, Massachusetts [17]. More recently, the first reproducing and established population in Connecticut was documented in Norwalk [19]. Their increasingly high densities in the Northeast in conjunction with their aggressive host-seeking behavior are cause for concern for host species, including humans.

*Amblyomma americanum* can transmit pathogens such as *Ehrlichia chaffeensis*, *E. ewingii*, *Francisella tularensis*, Heartland virus, and Bourbon virus [20]. More recently, it has also been associated with the development of alpha-gal meat allergy, which results in delayed anaphylaxis to an enzyme released during the digestive process of non-primate mammalian muscle tissue [21,22]. *Amblyomma americanum* seek out potential hosts in habitats similar to that of *I. scapularis*, woodlands with a dense understory shrub layer and ecotonal areas [23]. Additionally, the known range and the majority of human encounters have occurred in the milder coastal regions of the Northeast, suggesting that the range of *A. americanum* may be limited by climate-related variables. There have been several studies investigating the effects climate and climate change have had on the distribution of *A. americanum* throughout North America [24,25], but none have directly investigated overwintering survival.

We attempted to determine climatic variables that currently limit range expansion of *A. americanum* in the Northeast by monitoring adults to document the effects of geographically distinct winter weather conditions on their survival. We conducted the study over the course of three winters in both Connecticut and Maine to accommodate annual seasonal variability. Additionally, we manipulated natural insulating barriers including leaf litter and snow accumulation to determine which was more vital for their survival. The findings of our research will help determine if *A. americanum* can survive winters in inland and colder regions of the Northeast and what overwintering factors might predict and potentially aid in the management of this tick species.

## 2. Materials and Methods

### 2.1. Study Sites

The study took place over the course of three winters (2016–2017, 2017–2018, and 2018–2019, hereafter referred to as Years 1, 2, and 3) at two geographically distinct locations. The first site was at the Connecticut Agricultural Experiment Station’s Lockwood Farm in Hamden, Connecticut (41°24′ N, 72°54′ W). Woodlands consist of mature, upland hardwood stands with a mixture of oak typical of southern New England. The second site was located in Cape Elizabeth, Maine (43°34′ N, 70°13′ W). It is a second-growth deciduous and mixed dominant forest community typical of many post-agricultural woodlands in southern Maine. Further site vegetative and soil characteristics can be found in Linske et al. (2019) [7].

At both study sites, 24 plastic 4-L pots were placed in the ground in a linear orientation on 2 m spacing, approximately 20 cm deep, backfilled with the excavated soil to approximately 6 cm from the top. Three ~5 cm diameter holes were cut from the lid and bottom of each pot and covered in a fine mesh fabric to allow flow of both air and water. The intent was to make the confines of the pot as comparable to exterior conditions as possible. For each of the three years, all 24 pots at both locations housed three, ~5 cm long cylindrical plastic vials each containing three lab-reared adult male *A. americanum* (*n* = 1296). Holes were cut at both ends of each vial and covered with a fine mesh to contain adults. For all three years in Connecticut and for the first two years in Maine, there were also three lab-reared adult female *A. americanum* (*n* = 1080) in each of three vials; the State of Maine permitted import of males only in the third year of the study.

### 2.2. Treatment Assignments

We used a randomized block design to assign each of the 24 pots one of four treatments (6 replicates/treatment). Treatments consisted of no leaf removal and no snow removal (control), snow removal only (SR), leaf and snow removal (LRSR), and leaf removal only (LR). The control treatment was meant to mimic natural conditions with leaf litter over tick vials within the pot as well as on top of the closed lid. Any snow that accumulated over the course of the winter was not removed. The SR treatment similarly had leaf litter both in and over the tick pot, but was covered with a mesh cloth to facilitate snow removal without disturbing leaf litter approximately 4.0 m^2^ around the pot. The LRSR treatment had all leaf litter removed and likewise any snow accumulation removed from around the pot throughout the winters. Finally, the LR treatment consisted of tick pot and exterior void of leaf litter, but snow was not removed when it accumulated. Where appropriate, snow was removed immediately after each weather event that resulted in any accumulation and pots were checked and cleared regularly for leaf litter. Percent survival for each vial in each pot for each year was determined and recorded after removal in April.

### 2.3. Weather and Snow Data

A data logger (HOBO^®^ Pro v2 Temp/RH; Onset Computer Corp., Bourne, MA, USA) programmed to record temperature and relative humidity hourly was placed in each pot, adjacent to tick vials. In addition, three were affixed to metal stakes 1.0 m above ground level within the pot array that recorded ambient weather data. Tick vials and data loggers were deployed in early November and retrieved in mid-April, after snow melt. Snow was measured after each snow event at pot locations in Maine while in Connecticut, we used data from a monitored weather station on the same premises. For all analyses, we used temperature, relative humidity, and snow data from 15 December–28 February for each of the three winters. We used this interval because it was when snow and leaf removal treatments would have had the most combined influence on adult survival.

### 2.4. Statistical Procedures

#### 2.4.1. Three-Way ANOVA on Adult Survival

Percent survival of *A. americanum* in each vial in each pot for each year was determined and recorded after removal in April. We ran a three-way analysis of variance (ANOVA) on survival with treatment, sex, and year as factors in Connecticut for all three years and separately for Years 1 and 2 in Maine as there were no female data in Year 3.

#### 2.4.2. Three-Way ANOVA on Female and Male Survival

Based on results from the ANOVA for both sexes combined, we then conducted a three-way ANOVA on female survival with treatment, location, and year (Years 1 and 2 where comparable female data existed for both Connecticut and Maine) as factors. Because survival data were highly variable, we determined percent survival for each treatment for each year at each location which required square root-transformation to normalize. Similarly, we ran the same three-way ANOVA for male *A. americanum* survival for all three years at both locations combined with treatment, location, and year as factors. Male data were normal and did not require transformation.

#### 2.4.3. Weather Analyses

We averaged data for all 6 pots/treatment to determine mean temperature and relative humidity for each treatment at each location. We ran a Kruskal-Wallis one-way ANOVA on ranks with location (CT or ME) as the factor. Based on those results, we then ran Friedman one-way repeated measures ANOVA on ranks with treatment as the factor separately for data from Maine and Connecticut.

#### 2.4.4. Spearman Rank Order Correlation

We ran Spearman rank order correlation analyses for percent survival of males and females on mean hourly temperature/pot/location/year for the 15 December–28 February interval. We ran correlations for male survival and mean hourly temperature for all three years for both locations combined as well as each location separately. We also correlated female survival with mean hourly temperature for all three years in Connecticut and the first two years in Maine both combined and separately. SigmaPlot (Version 13, Systat Software, Inc., San Jose, CA, USA) statistical software was used for all statistical analyses. Tukey Honestly Significant Difference test with an alpha value of ≤0.05 was used for multiple comparison tests for all ANOVA analyses.

## 3. Results

### 3.1. Three-Way ANOVA on Adult Survival in Connecticut

Connecticut percent survival for adult *A. americanum* passed the Shapiro-Wilk Normality Test (*p* = 0.727) and the Brown-Forsythe equal variance (*p* = 1.000). There were no significant differences in survival between years (*F*_2,17_ = 0.647; *p* = 0.536) or treatment (*F*_3,17_ = 0.997; *p* = 0.418). However, there was a significant difference between sex (*F*_1,17_ = 5.395; *p* = 0.033); mean percent survival of females (64%) was significantly greater than males (53%).

### 3.2. Three-Way ANOVA on Adult Survival in Maine

Maine percent survival for adult *A. americanum* passed the Shapiro-Wilk Normality Test (*p* = 0.534) and the Brown-Forsythe equal variance (*p* = 1.000). There were no significant differences in survival between treatment (*F*_3,10_ = 2.547; *p* = 0.115) or sex (*F*_1,10_ = 2.334; *p* = 0.158). However, there was a significant difference between years (*F*_1,10_ = 35.308; *p* < 0.001). In Year 1, mean percent survival (8%) was significantly lower than Year 2 (43%).

### 3.3. Three-Way ANOVA on Female Survival

Square root transformed adult female survival data for Years 1 and 2 for Connecticut and Maine combined passed the Shapiro-Wilk Normality Test (*p* = 0.157) as well as the Brown-Forsythe test of equal variance (*p* = 1.000). No significant differences existed between treatments (*F*_3,10_ = 0.664; *p* = 0.593) but differences did differ between location (*F*_1,10_ = 13.642; *p* = 0.004) and year (*F*_1,10_ = 8.268; *p* = 0.017; Table 1). Overall percent survival was significantly lower in Maine (30%) than Connecticut (62%) and survival was significantly lower overall in Year 1 (35%) as compared to Year 2 (58%).

### 3.4. Three-Way ANOVA on Male Survival

Adult male survival data for Years 1, 2, and 3 for Connecticut and Maine combined passed the Shapiro-Wilk Normality Test (*p* = 0.309) as well as the Brown-Forsythe test of equal variance (*p* = 1.000). No significant differences existed between treatments (*F*_3,17_ = 1.316; *p* = 0.302; Table 2) but differences did exist between location (*F*_1,17_ = 57.932; *p* < 0.001) and year (*F*_1,17_ = 5.595; *p* = 0.014). Overall percent survival was significantly lower in Maine (17%) than Connecticut (53%) and survival was significantly higher in Year 2 (46%) than in both Years 1 (28%) and 3 (31%).

### 3.5. Weather Analyses

By Year 3, the data loggers’ relative humidity data were considered unreliable, most likely due to sustained exposure to high humidity conditions. As a result, we were unable to definitely compare relative humidity values between treatment types. Where reliable data did exist, mean daily relative humidity levels consistently exceeded 90% regardless of treatment, location, or year, which was consistent with the results of a similar study (Linske et al. 2019) [7]. However, mean daily temperature was significantly lower (χ^2^ = 473.870, df = 1, *p* < 0.001) in Maine (Mean = −1.33 °C) than Connecticut (Mean = 1.27 °C), regardless of treatment type (Table 3).

**Figure 1:**
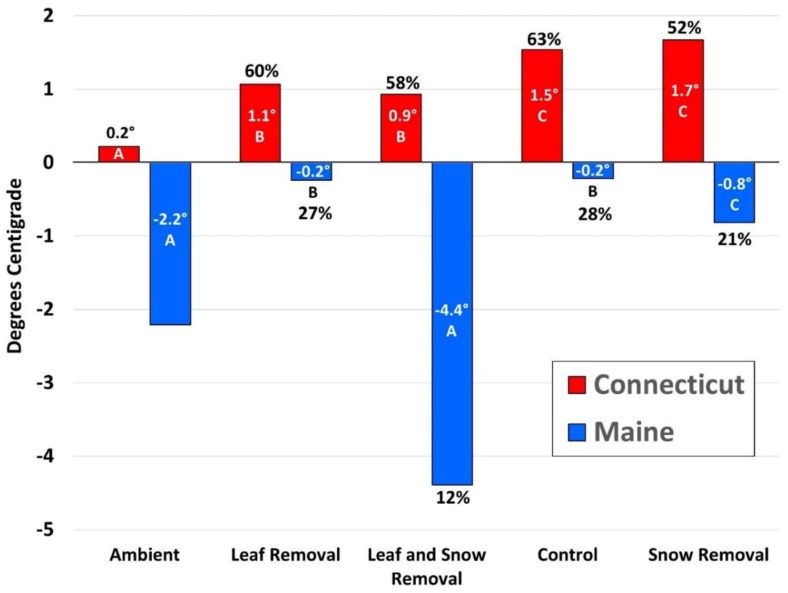
Mean hourly temperature (Centigrade) by treatment for Connecticut and Maine combined for Years 1, 2 and 3. Corresponding percent survival of adult *Amblyomma americanum* listed above and below Connecticut and Maine average temperatures, respectively. Mean temperature with the same letter assignment for each treatment within each state were not significantly different.

In Connecticut, significant differences existed in mean daily temperature between treatments (χ^2^ = 168.249, df = 4, *p* < 0.001) (Figure 1, Table 3). Ambient Connecticut mean daily temperatures were significantly lower than all four treatment types. In addition, mean daily temperature in the LRSR treatment was significantly lower than both control (*p* < 0.001) and the SR treatment (*p* < 0.001). Similarly, the LR treatment temperatures were significantly lower than both control (*p* < 0.001) and the SR treatment (*p* < 0.001). Treatments where leaf litter remained in both the control and SR treatment did not differ significantly (*p* = 0.057). Additionally, where leaf litter was removed in LRSR and LR treatments, mean daily temperature did not differ (*p* = 0.999; Figure 1).

In Maine, significant differences also existed in mean daily temperature between treatments (χ^2^ = 138.020, df = 4, *p* < 0.001; Figure 1). Differences existed in all treatment combinations except the LR treatment did not differ from control (*p* = 0.998) and the LRSR treatment did not differ from ambient (*p* = 0.980).

### 3.6. Survival and Mean Daily Temperature Correlation

While the LR and SR treatment combinations had a significant effect on mean temperature inside pots at both locations, treatment had no effect on survival at either location. However, survival was highly correlated with subfreezing temperatures, regardless of treatment. Male *A. americanum* survival was significantly positively correlated with mean temperature (*rs* = 0.624, *p* < 0.0001) for both locations for all three years combined and for all three years in Maine (*rs* = 0.447, *p* < 0.001). However, male survival was not significantly correlated with temperature in Connecticut (*rs* = −0.063, *p* = 0.607). For all three years in Connecticut and the first two years in Maine combined, female survival was also positively correlated with mean hourly temperature (*rs* = 0.470, *p* < 0.0001) as well as for the first two years in Maine (*rs* = 0.345, *p* = 0.018). Female survival was not significantly correlated with temperature for the three years in Connecticut (*rs* = −0.092, *p* = 0.452).

## 4. Discussion

In recent years, *A. americanum* has been expanding its range northward along coastal regions of the Northeast, establishing breeding populations in previously uninhabited areas [16,17,18,19]. It has been speculated that such populations are restricted to coastal areas, where winter weather conditions tend to be milder. As a result, the intent of this work was to determine what abiotic factors impact *A. americanum* survival, if they could survive winters further inland in a state with an existing, established coastal population (Connecticut), and if they could survive more severe winters in a northern, coastal location (Maine). We attempted to quantify driving factors of winter survival of *A. americanum* to be able to better predict its range expansion in response to a warming climate.

We found that adult *A. americanum* survival in Connecticut did not significantly differ by leaf and/or snow removal treatment type or year, but males did have a lower percent survival compared to females. For years 1 and 2 in Maine, adult survival did not differ by treatment or sex, but did differ by year. Female survival in Connecticut and Maine combined did not differ between treatments, but there was a significant difference between locations and years; Maine had lower survival success than Connecticut, and Year 1 had lower survival than Year 2. We ran the same analysis for males with similar results; no difference in treatments, but significantly greater survival in Connecticut compared to Maine and Years 1 and 3 had significantly lower percent survival compared to Year 2.

We initially predicted that survival would be highest in the control treatment and lowest where both leaf litter and snow was removed, as previously reported in a related study with *I. scapularis* nymphs [7]. However, this was not the case with adult *A. americanum*; survival did not differ by treatment assignment for all three years at each location. These results contradicted our original hypothesis resulting in further investigation as to whether treatment assignment had any effect on microclimate within individual pots. We found that in the virtual absence of snow (Table 4), leaf litter was essential for insulation in Connecticut as differences in mean daily temperatures existed between treatment combinations except for where leaf litter was manipulated similarly. In Maine, significant differences existed in mean temperatures between some treatment combinations, suggesting that both leaf litter and snow had a combined insulating effect. So while treatments had a documented effect on temperature, treatment type had no effect on adult *A. americanum* survival in either Maine or Connecticut. However, overall mean daily temperatures were significantly colder in Maine than Connecticut, regardless of treatment type (Figure 1, Table 3).

Because treatments had no effect on survival within year at each location, and because Maine winters were significantly colder with poorer survival, we correlated mean daily temperature with *A. americanum* survival within each pot at each location for each year by sex. Both male and female survival were significantly positively correlated with temperature, regardless of treatment in Connecticut and Maine combined. However, in Connecticut alone, neither male nor female survival were significantly correlated with temperature whereas in Maine, both male and female survival were significantly positively correlated with temperature. No combination of natural insulation in Maine could keep mean temperatures from falling below freezing (Figure 1; Table 3). This is in contrast to Connecticut where mean temperatures were above freezing in each treatment for all three years (Figure 1; Table 3). We believe the positive correlation for survival for both sexes in Maine can be attributed to sustained exposure to subfreezing temperatures (Figure 1), which has been shown to negatively impact survival rates both in the field and in laboratory settings [26].

Unlike species such as *H. longicornis* [27], *A. americanum* is non-parthenogenic and need to mate in order to reproduce [28]. It is likely that the combination of disproportionate survival in sexes and poor survival overall is limiting adult *A. americanum* establishment in the northern reaches of its range. While a small portion of adult *A. americanum* survived Maine winters, nearly 60% survived Connecticut winters. As a result, it would seem that it is merely a matter of time before breeding populations of *A. americanum* advance inland from established coastal areas in Connecticut while simultaneously continuing to advance northward along coastal New England as the climate continues to warm. It seems clear that southern Connecticut’s climate is currently conducive to *A. americanum* survival but that southern Maine is still too harsh for breeding populations to become established, though it was surprising that 22% of adult *A. americanum* survived Maine winters.

The ability of adult *A. americanum* to survive winters outside their historic natural range is likely due in part to an overall warming throughout the U. S., but particularly in the Northeast. In a recently published article in The Washington Post, Mufson et al. (2019) [29] depicted increases in mean winter temperatures (December, January, February) throughout the Northeast from 1895–2018. Central and coastal New Jersey, Suffolk County, NY, Fairfield and New London Counties, CT, Kent, Washington, and Newport Counties, RI, and Barnstable, Dukes, and Nantucket Counties, MA have all experienced some of the highest increases in mean winter temperatures in the Northeast (2.0–2.5 °C). Perhaps not coincidentally, the majority of these locations (Suffolk, Fairfield, Newport, Barnstable, Dukes, and Nantucket Counties) were recently documented to be the only locations in the Northeast (as well as New Haven County, CT) with established, breeding populations of *A. americanum* as reported by Molaei et al. (2019) [30]. In addition, Molaei et al. (2019) [30] indicated all counties in CT, the vast majority in MA, two in NH, and most of coastal Maine have reported the presence of *A. americanum*, but are not yet established. It is clear that as the climate in southern New England continues to warm, *A. americanum* populations will continue to expand and establish.

The combination of these recently reported findings in conjunction with our overwintering survival data indicate that while neither location has established populations due to harsher winter weather conditions, specifically mean temperature, individuals are still able to survive. With reports of breeding populations increasing and expanding throughout the Northeast [16,30], it is only a matter of time before temperatures rise to hospitable values that not only encourage survival but establishment of *A. americanum* populations as well. In response to the emerging threat of this vector species and its associated pathogens, studies such as ours can be utilized as part of a predictive analytic tool. With increasing temperatures throughout the Northeast, we should expect to see a positive correlation in survival and subsequent further establishment of *A. americanum* populations. In conjunction with other key predictors such as hospitable habitat, availability of hosts, and questing season factors such as temperature and humidity, we may be able to build off previous models [24,25] to better predict the expansion of the species. Comparable to the native *I. scapularis*, such information can be vital for developing more successful integrated tick management strategies that accommodate seasonal and annual variations in tick densities and distributions [31].

## 5. Conclusions

*Amblyomma americanum* is a nuisance tick species with aggressive host-seeking behavior, high population densities [15], and the known ability to overwhelm hosts [19]. In addition, their ability to cause human cases of ehrlichiosis, Bourbon virus infection, Heartland virus infection, southern tick-associated rash illness, spotted fever group rickettsia, tularemia, and red meat allergy makes them a species of concern [21,32,33,34]. We need to be prepared for their expansion into more northern regions as temperatures warm allowing for increased survival where mean sub-soil winter temperatures remain above freezing. Studies such as ours provide valuable information that could aid in predicting the future density and distribution of the species. Predictive analytics involving such variables in conjunction with adaptive management will play a necessary role in controlling, reducing, or possibly eliminating future threats incurred by *A. americanum* range expansion in the U.S.

## Figures and Tables

**Table 1:** Adult female *Amblyomma americanum* (*n* = 54/treatment/location/year) survival for Connecticut Years 1, 2, and 3 and Maine for Years 1 and 2 by treatment (LR = leaf removal only, LRSR = leaf and snow removal, SR = snow removal only, Control = no leaf or snow removal). No significant differences existed between treatments.

Treatment	Connecticut			Maine	
	Year 1	Year 2	Year 3	Year 1	Year 2
Control	56%	69%	78%	7%	73%
SR	32%	52%	69%	9%	41%
LR	83%	63%	65%	9%	62%
LRSR	69%	73%	59%	11%	27%
Mean	60%	64%	68%	9%	51%

**Table 2:** Adult male *Amblyomma americanum* (*n* = 54/treatment/location/year) survival for Connecticut Years 1, 2, and 3 and Maine for Years 1 and 2 by treatment (LR = leaf removal only, LRSR = leaf and snow removal, SR = snow removal only, Control = no leaf or snow removal). No significant differences existed between treatments.

Treatment	Connecticut			Maine		
	Year 1	Year 2	Year 3	Year 1	Year 2	Year 3
Control	46%	69%	61%	13%	40%	18%
SR	47%	47%	65%	7%	35%	6%
LR	56%	59%	37%	2%	56%	4%
LRSR	48%	49%	50%	2%	11%	7%
Mean	49%	56%	53%	6%	36%	9%

**Table 3:** Mean hourly temperature (°C) for Connecticut and Maine for Years 1, 2, and 3 by treatment (LR = leaf removal only, LRSR = leaf and snow removal, SR = snow removal only, Control = no leaf or snow removal).

Treatment	Connecticut			Maine		
	Year 1	Year 2	Year 3	Year 1	Year 2	Year 3
Ambient	0.83	−1.17	−0.40	−1.70	−4.19	−3.34
Control	2.71	0.96	1.79	−0.36	−0.15	−0.86
SR	2.82	1.10	1.98	−0.65	−1.55	−1.35
LR	2.22	0.73	1.30	−0.48	−0.04	−0.99
LRSR	2.15	0.59	1.45	−0.86	−1.85	−1.54
Mean	2.14	0.44	1.22	−0.81	−1.56	−1.62

**Table 4:** Total number of non-snow days compared to days with snow for both Connecticut and Maine over the course of three winters. For days of snow, average monthly snow accumulation and total average snow accumulation are also reported.

Location	Year	Month	# of No-Snow Days	# of Snow Days	Avg. Monthly Snow Accumulation (cm)
CT	1	Dec	15	2	1.0
CT	1	Jan	26	5	9.2
CT	1	Feb	16	12	18.2
CT	2	Dec	12	5	2.0
CT	2	Jan	16	15	11.4
CT	2	Feb	25	3	7.2
CT	3	Dec	16	1	1.0
CT	3	Jan	25	6	2.0
CT	3	Feb	21	7	1.0
	Total		172	56	Mean = 5.9
ME	1	Dec	0	17	10.7
ME	1	Jan	6	25	26.4
ME	1	Feb	0	28	45.6
ME	2	Dec	0	17	20.2
ME	2	Jan	0	31	57.4
ME	2	Feb	0	28	16.1
ME	3	Dec	13	4	5.1
ME	3	Jan	5	26	10.1
ME	3	Feb	0	28	22.0
	Total		24	204	Mean = 23.7
